# Diagnostic Performance of Artificial Intelligence-Centred Systems in the Diagnosis and Postoperative Surveillance of Upper Gastrointestinal Malignancies Using Computed Tomography Imaging: A Systematic Review and Meta-Analysis of Diagnostic Accuracy

**DOI:** 10.1245/s10434-021-10882-6

**Published:** 2021-11-11

**Authors:** Swathikan Chidambaram, Viknesh Sounderajah, Nick Maynard, Sheraz R. Markar

**Affiliations:** 1 Department of Surgery and Cancer, Imperial College London, London, UK; 2 Institute of Global Health Innovation, Imperial College London, London, UK; 3 Department of Surgery, Churchill Hospital, Oxford University Hospitals NHS Trust, Oxford, UK; 4 Department of Molecular Medicine and Surgery, Karolinska Institutet, Stockholm, Sweden

## Abstract

**Background:**

Upper gastrointestinal cancers are aggressive malignancies with poor prognosis, even following multimodality therapy. As such, they require timely and accurate diagnostic and surveillance strategies; however, such radiological workflows necessitate considerable expertise and resource to maintain. In order to lessen the workload upon already stretched health systems, there has been increasing focus on the development and use of artificial intelligence (AI)-centred diagnostic systems. This systematic review summarizes the clinical applicability and diagnostic performance of AI-centred systems in the diagnosis and surveillance of esophagogastric cancers.

**Methods:**

A systematic review was performed using the MEDLINE, EMBASE, Cochrane Review, and Scopus databases. Articles on the use of AI and radiomics for the diagnosis and surveillance of patients with esophageal cancer were evaluated, and quality assessment of studies was performed using the QUADAS-2 tool. A meta-analysis was performed to assess the diagnostic accuracy of sequencing methodologies.

**Results:**

Thirty-six studies that described the use of AI were included in the qualitative synthesis and six studies involving 1352 patients were included in the quantitative analysis. Of these six studies, four studies assessed the utility of AI in gastric cancer diagnosis, one study assessed its utility for diagnosing esophageal cancer, and one study assessed its utility for surveillance. The pooled sensitivity and specificity were 73.4% (64.6–80.7) and 89.7% (82.7–94.1), respectively.

**Conclusions:**

AI systems have shown promise in diagnosing and monitoring esophageal and gastric cancer, particularly when combined with existing diagnostic methods. Further work is needed to further develop systems of greater accuracy and greater consideration of the clinical workflows that they aim to integrate within.

Esophageal cancer is an aggressive cancer with a mean estimated 5-year survival rate of 35–45%, even after treatment with curative intent.^1, 2^ The reported survival rate in advanced-stage disease drops further to 5–10% and can be attributed to the malignancy’s insidious onset and aggressive tumor biology that often favors recurrence.^3–5^ Similarly, gastric cancer has a poor 5-year survival rate and is still the third leading cause of malignancy-related death worldwide.^6^ A number of investigations, such as computed tomography (CT) scans, positron emission tomography (PET) scans, endoscopic ultrasound (EUS), and endobronchial ultrasound (EBUS), are utilized in the diagnostic and staging pathway of esophagogastric (EG) malignancy, with CT being the most commonly used of those that are noted.^7^ Unlike colorectal, hepatocellular, and pancreatic cancers, there is no reliable biomarker that can be tested and tracked non-invasively for diagnostic or surveillance purposes in esophageal and gastric cancers.^8–10^ Consequently, patients are often reliant on radiological investigations for diagnosis with staging, detection of recurrence, and monitoring response to treatment.^7^ These workflows necessitate both timely and expert radiological interpretation, a requirement that is often difficult to achieve given busy clinical work schedules and a lack of expertise outside tertiary oncological centers. As such, there has been increasing calls to explore the use of AI-centred diagnostic systems to alleviate this issue.

In the context of medical diagnostics, AI is the use of a system to mimic human cognition in the comprehension, analysis, and presentation of medical data.^11–13^ This is often achieved using machine learning (ML), which is a specialized sub-field within AI that improves the performance of systems through repetitive experience. For example, in EG cancers, ML has been used extensively by AI systems to understand endoscopy images and enhance the interpretation of solely operator-dependent endoscopy.^14–16^ Naturally, the next step will be the integration of AI into the major imaging modalities used in the management of EG cancers, specifically CT scans. Typically, this involves the high-throughput extraction of large quantities of data from the images and is a technique termed as radiomics. Radiomics is an emerging field using a non-invasive approach to extract numerous quantitative features from medical images, especially parameters not visible to the naked human eye or quantifiable by routine analysis.^17, 18^ Specifically, with CT scans, radiomics offers the unique advantage of combining ML to acquire images; segment images into regions of interest (ROIs) or volumes of interest (VOIs); extraction of quantitative imaging features from ROIs and VOIs; and, lastly, constructing and validating models. Recently, there has been an increase in work reporting on the combined or individual use of AI or radiomics to diagnose or monitor EG cancers. This review aims to summarize the potential applicability of AI diagnostic systems in the diagnosis and surveillance of esophageal and gastric cancers.

## Methods

Literature search methods, inclusion and exclusion criteria, outcome measures, and statistical analysis were defined according to the Preferred Reporting Items for Systematic Reviews and Meta-Analyses (PRISMA) guidelines.^19^ Patients were not involved in the conception, design, analysis, drafting, interpretation, or revision of this research, hence ethical approval was not required and was thus not sought for this study.

### Literature Search

The following databases were searched: MEDLINE (from 1946 until the first week of April 2021) via OvidSP; MEDLINE In-Process and other non-indexed citations (latest issue) via OvidSP; Ovid EMBASE (from 1974 to the latest issue); and Scopus (from 1996 until the present). The last search was performed on 15 April 2021. Search terms used several strings that were linked by standard modifiers in the following order: ‘machine learning’, ‘artificial intelligence’, ‘radiomic’, ‘AI’ OR ‘ML’, as well as ‘esophageal cancer’, ‘esophageal squamous cell cancer’, ‘esophageal adenocarcinoma’, ‘ESCC’, ‘EAC’, ‘esophageal malignancy’, ‘upper gastrointestinal cancer’, OR ‘upper GI cancer’. Additionally, the references of included articles were hand-searched to identify any additional studies.

### Selection and Quality Assessment of Studies

Articles were screened for eligibility by SC and VS, and, where conflict arose, a third co-author (SRM) was consulted. Studies were included if they had incorporated the use of AI-centred systems in CT imaging for evaluating both esophageal and gastric cancers. Studies with diagnostic, prognostic, and monitoring intents were included. Studies were excluded if they did not evaluate ML, used imaging modalities other than CT, did not include patients with esophageal or gastric cancers, had incomplete data on outcome measures, were not written in the English language, had sample sizes fewer than 30 patients, or had incompatible designs, including letters, comments and reviews. Studies were assessed for robustness of methodology using the Quality Assessment Tool for Diagnostic Accuracy Studies 2 (QUADAS-2), which comprises four domains covering patient selection, index test, reference standard, and flow of patients through the study and timing of the index test(s) and reference standard. Each domain is evaluated in terms of the risk of bias, and the first three domains are also assessed for any concerns regarding applicability. In doing so, this highlights aspects of the study design that may be exposed to bias.

### Statistical Analysis

All statistical analyses were performed using STATA/SE version 16.0 (StataCorp LLC, College Station, TX, USA). The overall pooled estimate of sensitivity and specificity, with their corresponding 95% confidence intervals (CIs), was calculated using the random-effects model with the metandi command in STATA/SE. Sensitivity was defined as the proportion of patients with esophageal cancer who were correctly confirmed by AI, while specificity was defined as correctly identifying patients without the disease. Forest plots were used to visualize the variation of the diagnostic parameter effect size estimates with 95% CI and weights from the included studies.

## Results

### Study Selection

**Fig. 1:**
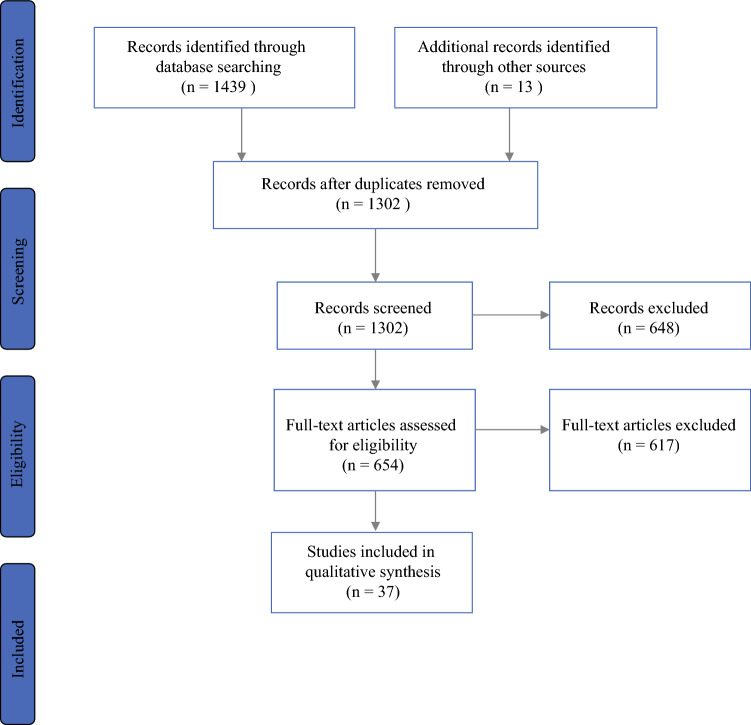
PRISMA diagram showing the sequence of the study screening and selection process. *PRISMA* Preferred Reporting Items for Systematic Reviews and Meta-Analyses

The database search yielded a total of 1439 studies, of which 137 duplicates were removed. Titles and abstracts of the remaining 1302 studies were screened for eligibility and 648 studies were removed. A further 617 studies were excluded after full-text review due to incompatible outcome measures, study design, or small sample sizes of fewer than 30 patients (Fig. 1). Thirty-seven studies that described the use of ML (a branch of AI) platforms for the diagnosis and surveillance of esophageal and gastric cancers were included in this study (Table 1).

**Table 1:** Characteristics of the included studies

Author	Year	Sample size	Design	Purpose	Condition	Radiomic/AI approach	Sensitivity	Specificity	AUC	Accuracy	Results
Ba-Ssalamah	2013	67	Retrospective	Diagnostic	Gastric cancer	ROI					Differentiate between adenocarcinoma and lymphoma with a misclassification rate of 3.1%
Dong	2019	554	Retrospective	Diagnostic/staging	Gastric cancer	ROI			0.92–0.95		Effective model for prediction of occult metastasis
Dong	2020	730	Retrospective	Diagnostic/staging	Gastric cancer	ROI					Deep learning-based radiomic nomogram had good predictive value for LNM in LAGC
Feng	2019	490	Retrospective	Diagnostic/staging	Gastric cancer	ROI			0.76–0.82		Differentiate between node status
Feng	2021	189	Retrospective	Diagnostic/staging	Gastric cancer	ROI					Differentiate primary gastric lymphoma from Borrmann type IV gastric cancer
Jin	2021	572	Retrospective	Diagnostic/staging	Gastric cancer		0.743	0.936	0.876		Prediction of lymph node metastasis in gastric cancer
Liu	2017	80	Prospective	Diagnostic/staging	Gastric cancer	VOI	72	81	0.79	74.0	Differentiate between node status with 74% accuracy
Liu	2017	87	Prospective	Diagnostic/staging	Gastric cancer	VOI	76	86	0.8		Differentiate between node status
Liu	2018	64	Retrospective	Diagnostic/staging	Gastric cancer	VOI	86	75	0.82	81.0	Differentiate between vascular status with 81% accuracy
Liu	2017	107	Retrospective	Diagnostic/staging	Gastric cancer	ROI					High correlation between histology and radiomic features (r = −0.231 to 0.324)
Ma	2017	70	Retrospective	Diagnostic	Gastric cancer	VOI	70	100	0.9	87.0	Differentiate between adenocarcinoma and lymphoma with an accuracy of 86%
Meng	2021	539	Retrospective	Diagnostic	Gastric cancer	ROI					2D radiomic features are better than 3D features at LNM and lymphovascular prediction, as well as staging cancers
Wang	2020	187	Retrospective	Diagnostic	Gastric cancer	VOI			0.904		Predictive model for distinguishing intestinal-type gastric cancer
Wang	2020	515	Retrospective	Diagnostic	Gastric cancer	ROI	85	72.7	0.814	83.4	CT-based radiomics nomogram provides a promising and more effective method to yield high accuracy in the identification of No. 10 LNMs in APGC patients
Wang	2020	353	Retrospective	Diagnostic	Gastric cancer	ROI	353				MDCT radiomic signature has the potential to predict 2-year disease-free survival
Wang	2021	159	Retrospective	Diagnostic	Gastric cancer	ROI					Radiomic nomograms have favorable predictive accuracy in predicting No. 3 LNM in T1-2 GC, and LNM in No. 4 LNs
Zhang	2017	78	Retrospective	Diagnostic/staging	Gastric cancer	VOI			<0.7		Poor ability to differentiate between grades
Giganti	2017	34	Retrospective	Surveillance	Gastric cancer	VOI					Effective model for prediction of response to chemotherapy
Giganti	2017	56	Retrospective	Surveillance	Gastric cancer	VOI					Effective model for prediction of response to curative resection
Hou	2018	43	Retrospective	Surveillance	Gastric cancer	VOI			0.686–0.728		Prediction of response to radiotherapy with AUCs up to 0.728
Jiang	2018	1591	Retrospective	Surveillance	Gastric cancer	ROI					Model is predictive of disease-free survival and overall survival
Jiang	2018	214	Retrospective	Surveillance	Gastric cancer	VOI					Effective model to prediction of survival and response to chemotherapy
Jiang	2019	1689	Retrospective	Diagnosis/staging	Gastric cancer	ROI					Radiomics signature was significantly associated with pathological LN stage and hence a good predictor of LNM
Li	2018	181	Retrospective	Surveillance	Gastric cancer	ROI, VOI					More effective model than clinical parameters in predicting prognosis post-resection
Li	2018	30	Retrospective	Surveillance	Gastric cancer	VOI			0.722		Effective model to predict non-responders to chemotherapy
Shin	2021	410	Retrospective	Surveillance	Gastric cancer	ROI					Radiomics-based model on preoperative CT images may improve RFS prediction and high-risk stratification in the preoperative setting of LAGC
Yoon	2016	26	Retrospective	Surveillance	Gastric cancer	ROI			0.75–0.77		Effective model for prediction of poorer survival outcomes
Zhang	2020	669	Retrospective	Surveillance/screening	Gastric cancer	ROI			0.806–0.831		Potential tool for prediction of response to chemotherapy
Chang	2021	200	Retrospective	Diagnostic/staging	EAC	ROI	0.835	0.839			11-feature radiomic model can differentiate between T3 and T4a stages of EGJ adenocarcinoma
Wang	2017	131	Retrospective	Diagnostic	EAC and ESCC	SVM			0.887		Support vector machine model of CT images can help diagnose LNM in esophageal cancer with preoperative chemotherapy
Takeuchi	2021	457	Retrospective	Diagnostic	EAC and ESCC	CNN	0.72	0.91		0.842	Effective model for the diagnosis of cancer
Foley	2018	403	Retrospective	Surveillance	EAC (316); ESCC (87)	ATLAAS					Prognostic model can risk-stratify patients in staging
Hu	2020	231	Retrospective	Surveillance	EAC and ESCC	ROI					Peri- and intra-tumoral radiomics features can predict tumor response to nCRT
Jin	2020	94	Retrospective	Surveillance	EAC and ESCC	XGBoos			0.479–0.541	68.9–70.8	Combining dosimetric and radiomic features improves the predictive accuracy of models
Li	2020	57	Retrospective	Surveillance	ESCC	ROI	0.727	0.875	0.454	0.815	Radiomics models can accurately detect the hetereogeneity in late-stage ESCC
Rishi	2020	68	Retrospective	Surveillance	EAC and ESCC	VOI			0.87	0.77	Composite CT/PET radiomics model was highly predictive of pCR following nCRT

*AI* artificial intelligence, *AUC* area under the curve, *EAC* esophageal adenocarcinoma, *ESCC* esophageal squamous cell carcinoma, *ROI* region of interest, *VOI* volume of interest, *SVM* support vector machine, *LNM* lymph node metastasis, *LAGC* locally advanced gastric cancer, *2D* two-dimensional, *3D* three-dimensional, *APGC* α-fetoprotein-producing gastric cancer, *MDCT* multidetector computed tomography, *GC* gastric cancer, *LNs* lymph nodes, *CT* computed tomography, *RFS* recurrence-free survival, *EGJ* esophageal gastric junction, *nCRT* neoadjuvant chemoradiotherapy, *PET* positron emission tomography, *pCR* pathologic complete response, *ATLAAS* Automatic decision tree learning algorithm for advanced segmentation

### Quality Appraisal

Assessment of studies using the QUADAS-2 tool showed a low level of bias among the studies (Table 2). The risk of bias and concerns on their applicability was low across most domains. Some risk of bias was present due to the heterogeneity of the patients included; however, in most studies, there was little reporting of the sensitivity and specificity of the ML algorithms used.

**Table 2:** Characteristics of image acquisition and processing using AI and/or radiomic approaches

Author, year	Image acquisition protocol	Imaging parameters and segmentation	AI program/radiomic features extracted	Texture analysis software
Ba-Ssalamah [24]	4 scanners; CT scans during the arterial and portal venous phases and reconstructed with a soft tissue kernel	Tube voltage, 120 kV; tube current, 230 mAs; collimation, 16 mm × 0.75 mm; reconstruction orientation, transverse; reconstruction section thickness, 1 mm (arterial phase) and 4 mm (portal-venous phase) with 2 mm increments; and matrix, 512 × 512 Segmentation: ROI	First-order statistics; second-order GLCM, RLM statistics; wavelet transformed statistics	MaZda 4.6; LDA in combination with k nearest-neighbor classification
Dong, 2019	Several scanners; pretreatment PP CT	Tube voltage, 120 kV; tube current, 120–550 mAs; collimation, 64 × 0.625 mm; reconstruction orientation, transverse; reconstruction section thickness, 1.25–5 mm (portal-venous phase) with 2 mm increments; and matrix, 500 × 500 Segmentation: ROI	3D shape and size features; first-order statistics; second order GLCM and RLM statistics	ITK-SNAP software
Dong, 2020	Several scanners; pretreatment PP CT	Tube voltage, 120 kV; tube current, 120–550 mAs; collimation, 64 × 0.625 mm; reconstruction orientation, transverse; reconstruction section thickness, 1.25–5 mm (portal-venous phase) with 2 mm increments; and matrix, 500 × 500 Segmentation: ROI	3D shape and size features; first-order statistics; second-order GLCM and RLM statistics	ITK-SNAP software
Feng, 2019	1 scanner; preoperative PP CT	Segmentation: ROI	First-order statistics, second-order GLCM statistics	–
Feng, 2021	1 scanner; preoperative PP CT	Segmentation: ROI	First-order statistics, second-order GLCM statistics	–
Liu, 2017	2 scanners; arterial and portal venous phase CT images	Tube voltage 120 kVp, tube current 250–350 mA, slice thickness 5 mm, slice interval 5 mm, field of view 35–50 cm, matrix 512 × 512, rotation time 0.7 s and pitch 1.375 Segmentation: ROI	First-order statistics	In-house software (Image Analyzer 1.0, China)
Liu, 2017	1 scanner; pretreatment ADC map	Respiratory triggered turbo spin-echo sequence without fat saturation (repetition time msec/echo time msec, 1210–1220/70; matrix, 256 × 198; section thickness, 4 mm; gap, 1 mm; number of sections, 32–36; field of view, 36 cm; sensitivity encoding factor, 3.0; number of signal averaged, 1) Segmentation: VOI	First-order statistics	In-house software (Image Analyzer 1.0, China)
Liu, 2018	1 scanner; pretreatment ADC map	Segmentation: VOI	First-order statistics	In-house software (Image Analyzer 1.0, China)
Liu, 2017	1 scanner; pretreatment ADC map	Segmentation: VOI	First-order statistics	In-house software (Image Analyzer 1.0, China)
Ma [25]	2 scanners; 25–30 s (arterial phase), 60 s (portal phase), and 180 s (delayed phase)	120 kVp; 130 mAs; rotation time, 0.5 s; detector collimation, 64 × 0.625 mm or 8 × 0.625 mm; field of view, 350 × 350 mm; matrix, 512 × 512; and reconstruction section thickness, 1.25 mm Segmentation: VOI	First-order statistics, shape- and size-based features (including tumor volume), texture features, wavelet features MATLAB program used	3D Slicer software
Wang, 2020	1 scanner; preoperative PP CT	–	Final radiomic features were composed of eight groups according to the IBSI C-index, AUC, and DCA, comparison of the three prognostic models (radiomic signature, radiomic nomogram, and TNM staging model)	ITK-SNAP
Wang, 2020	1 scanner; preoperative PP CT	–		ITK-SNAP
Giganti [27]	1 scanner; unenhanced, late arterial and portal venous phases	64 detector rows; beam collimation: 64 × 0.62; pitch: 0.983; kVp/effective mA: 120/300; slice thickness: 2 mm; gap: 1 mm. Segmentation: VOI	3D shape and size features; first-order statistics, second-order GLCM and RLM statistics MATLAB program used	MIPAV, version 7.2.0
Giganti [28]	1 scanner; unenhanced, late arterial and portal venous phases	64 detector rows; beam collimation: 64 × 0.62; pitch: 0.983; kVp/effective mA: 120/300; slice thickness: 2 mm; gap: 1 mm Segmentation: VOI	First-order statistics, second-order GLCM and RLM statistics MATLAB program used	MIPAV, version 7.2.0
Hou [32]	1 scanner; pretreatment AP CT	Tube voltage, 120 kVp; tube current, 200–250 mAs; rotation time, 0.75 s; pitch, 0.9; matrix, 512 × 512; convolution kernel, standard	First-order statistics, second-order GLCM and RLM, NGTDM, GLSZM statistics	3D Slicer software
Li [29]	Arterial and venous phase	512512; layer thickness was 5 mm, layer spacing was 5 mm, 120 Kv; B31f reconstruction function, respectively	Receiver operator curve analysis was conducted to evaluate the performance of the tumor grade diagnosis model	A.K. software (Analysis Kit) and ITK-SNAP
Yoon [33]	3 scanners; pretreatment PP CT	Helical scan data were acquired using 16 × 1.5, 64 × 0.625, or 128 × 0.625 mm collimation; a rotation speed of 0.5 s; a pitch of 1.25, 0.641, or 0.993; and a kvP of 120 kVp). Using an automatic tube current modulation technique (Dose-Right; Philips Medical Systems), effective mAs ranged from 69 to 379 mAs. Transverse and coronal section datasets were reconstructed with 4-mm thick sections at 3-mm increments Segmentation: ROI	First-order statistics, second-order GLCM statistics	
Wang [22]	Pretreatment PP CT	Chest unenhanced CT scans were acquired with 0.625 mm collimation, 120–140 kVp, and 300–350 mAs	Least squares SVM modeling	MATLAB
Takeuchi [20]	Pretreatment PP CT	Tube voltage, 120 kVp; tube current, 100–750 mA; and pitch, 1.375:1	CNN-based model using training	
Foley [21]	Pretreatment PP CT	CT images were acquired in a helical acquisition with a pitch of 0.98 and tube rotation speed of 0.5 s. Tube output was 120 kVp with output modulation between 20 and 200 mA. Matrix size for the CT acquisition was 512 × 512 pixels with a 50-cm field of view		ATLAAS segmentation
Rishi, 2020	Pretreatment PP CT	Image resolution was 128 9 128 pixels, with voxel dimensions of 5.47 9 5.47 9 3.27 mm, and slice thickness of 3.27 mm. CT images were reconstructed using 3D CT attenuation correction with standard filtered back-projection reconstruction 512 9 512 in 50–70 cm FOV Segmentation: VOI	126 features were extracted from both PET and CT scans, including intensity (27 features), shape (11 features), GLCM (40 features), GLRLM (17 features), GLSZM (12 features), NGTDM (11 features), and FD (8 features)	Mirada RTx

*AI* artificial intelligence, *CT* computed tomography, *ROI* region of interest, *LDA* linear discriminant analysis, *3D* three-dimensional , *VOI* volume of interest, *IBSI* image biomarker standardization initiative, *AUC* area under the receiver operating characteristic curve, *DCA* decision curve analysis, *SVM* support vector machine, *ATLAAS* Automatic Decision Tree Learning Algorithm for Advanced Segmentation, *PET* positron emission tomography, *GLCM* gray-level co-occurrence matrix, *GLRLM* gray-level run-length matrix, *RLM* run-length matrix, *GLSZM* gray-level size-zone matrix, *NGTDM* neighborhood gray-tone difference matrix, *FD* fractal dimension, *FOV* field of view, *3D* three-dimensional

**Table 3:**
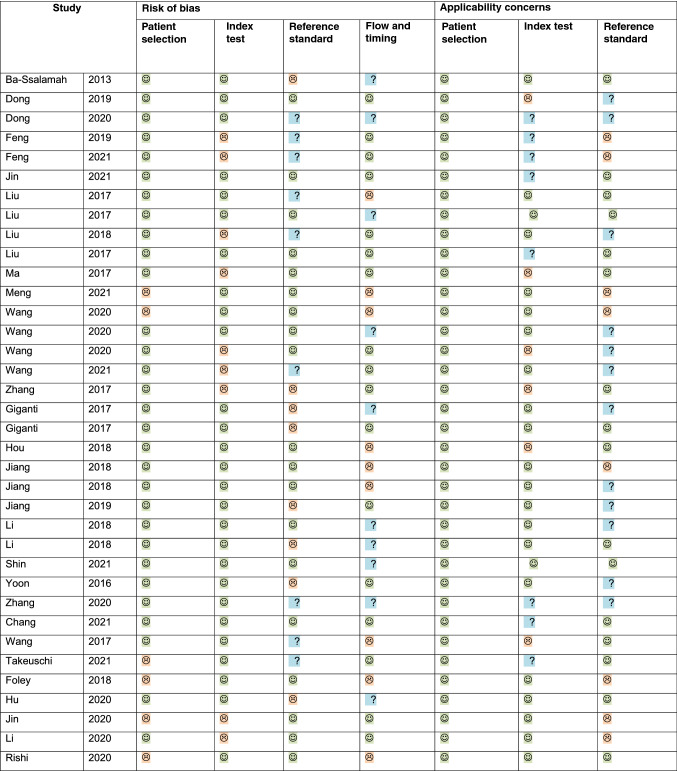
QUADAS assessment of studies included for risk of bias and applicability

### Use of Machine Learning and Radiomics in the Management of Esophageal Cancer

Takeuchi et al. reported diagnostic accuracy of 84% (sensitivity 71.7%; specificity 90.0%) in detecting stage T1–T5 esophageal cancer in 46 patients.^20^ One study looked at the prognosis of patients with esophageal cancer, in which Foley et al. reported six variables to be predictive of overall survival in their work of 405 patients.^21^ Two studies evaluated the use of ML to assess response to chemoradiotherapy for esophageal cancers. The model developed by Wang et al. to evaluate the scan of 131 patients who underwent neoadjuvant chemotherapy diagnosed lymph node metastasis better than the preoperative short axis size of the largest lymph node on CT, with an area under the curve (AUC) of 0.887.^22^ Jin et al. combined a radiomics and dosimetric approach and reported an AUC of 0.708 in predicting the treatment response of patients with esophageal cancer who underwent chemoradiotherapy.^23^

### Use of Machine Learning and Radiomics in the Management of Gastric Cancer

Two studies investigated the use of radiomics in diagnosing gastric cancer, specifically in differentiating gastric cancer from other gastric lesions.^24, 25^ In their study evaluating VOI-based textural features on preoperative arterial phase and portal phase scans of 95 patients, Ba-Ssalamach et al. differentiated gastric adenocarcinoma with an error rate as low as 3.1%.^24^ Two studies reported that there was little correlation between radiomic features and histological grades, with AUCs below 0.7,^9, 10^ while five studies evaluated images for lymph node status, vascular invasion, and occult peritoneal metastasis, with AUCs as high as 0.941.^11–15^ Of the included studies, two studies evaluated the use of AI for prognosis after surgical resection for gastric cancers. Li et al. extracted 273 features from each ROI and 485 features from each VOI, and used the least absolute shrinkage and selection operator (LASSO) method to predict overall survival, although the results were not promising in their test set.^26^ In contrast, Giganti et al. extracted 107 features from each VOI that were significantly associated with a negative overall survival in patients with resectable gastric cancer.^27^ Four studies also investigated the use of AI for predicting response to neoadjuvant chemotherapy. Giganti et al. determined 14 features in pretreatment arterial phase images that were significantly different between responders and non-responders, while another study by Li et al. showed similar results with portal venous phase images.^28, 29^ In their multicenter study, Jiang et al. identified potential predictors from portal venous phase scans of 1591 patients that were significantly different between responders and non-responders to neoadjuvant chemotherapy and predictive of disease-free survival.^30, 31^ Two studies evaluated the response to targeted immunotherapy with trastuzumab or radiotherapy. Hou et al. showed that radiomic signatures can predict response to radiotherapy with an AUC of 0.749, while Yoon et al. reported AUCs of 0.75–0.77 in their small pilot study of 26 cases of HER2-positive gastric cancer treated with trastuzumab.^32, 33^

### Artificial Intelligence as a Diagnostic and Monitoring Tool: Quantitative Analysis

**Fig. 2:**
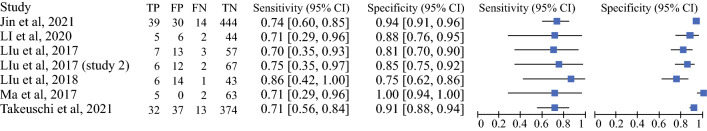
Forest plot of diagnostic accuracy for machine learning platforms. *TP* true positive, *FP* false positive, *FN* false negative, *TN* true negative, *CI* confidence interval

**Fig. 3:**
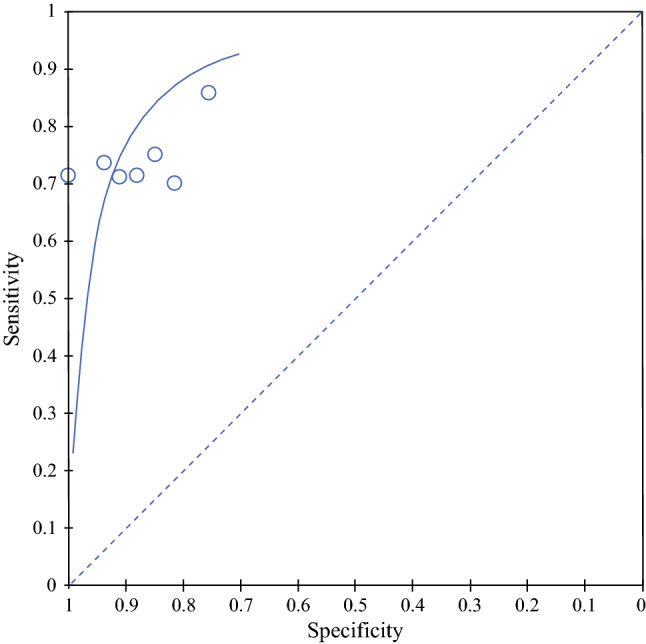
Summary receiver operating characteristic curve for diagnostic accuracy for machine learning platforms

Six studies involving 1352 patients provided sufficient data of true positive, true negative, false positive, and false negative rates for the calculation of sensitivity and specificity. Of these studies, four studies assessed its utility in gastric cancer diagnosis, one study assessed its utility for diagnosing esophageal cancer, and one study assessed its utility for surveillance (Table 1). The pooled sensitivity and specificity were 73.4% (64.6–80.7) and 89.7% (82.7–94.1), respectively, as visualized on the forest plot and summary receiver operating characteristic curve (Figs. 2 and 3).

## Discussion

Our systematic review shows that the application of radiomics and AI for the diagnosis and surveillance of upper gastrointestinal tract malignancies is promising, despite being in its nascency. The included radiological studies show that AI can be potentially used to diagnose cancers, differentiate malignancies from benign lesions, and detect occult disease. AI systems may also be used for staging disease, determining if surgery will improve survival outcomes in patients with resectable disease, and in predicting whether patients will respond to adjuvant or neoadjuvant chemoradiotherapy. Our paper also highlights the different AI platforms available for these purposes and captures their breadth.

The typical patient undergoes several CT scans during their journey, with diagnosis as the primary aim. Combining radiomics and AI to current scans will enable clinicians to simultaneously predict how they will respond to treatment and also assess how they have responded to treatment. In other cancers, radiomic data have provided support to genomic data in generating a prognostic signature that exceeds the accuracy of traditional TNM staging.^34^ Given that there is a direct correlation between histopathological response of patients who underwent chemoradiotherapy and the overall survival rate, the ability to assess clinical response will be useful in adjusting the dose and regimes of chemoradiotherapy.^35, 36^ Our paper has included at least one study using radiomics or AI to assess the response to surgery, chemotherapy, radiotherapy and immunotherapy, and all report high performance; however, there is still scarce evidence to add support to existing studies described here.

AI can also help in overcoming any technical limitations faced by traditional imaging. For example, Jin et al. combined radiomic and dosimetric analyses to overcome the artefacts in wall thickness created by the regular peristaltic waves of contraction.^23^ In another study, Ding et al. showed that their models detected occult peritoneal metastasis more accurately than conventional CT scans. Previous studies including the Worldwide Esophageal Cancer Collaboration have reported that survival decreases with the presence of lymph node metastases, and imaging examinations are often the first-line investigations for assessing most lymph node statuses in esophageal cancer.^37–39^ However, the accuracy of CT in diagnosing the N stage of esophageal cancer was just 59%.^40^ Most clinicians use a size criterion of 1 cm to differentiate between benign and malignant enlargement of lymph nodes but this only has a sensitivity of 30–60% and a somewhat higher specificity of 60–80%.^41–43^ In their study, Wang et al. showed that support vector machine (SVM) models have better diagnostic capability for lymph node metastasis than the traditional LN size criteria.^22^ Furthermore, Bollschweiler et al. used a different ML methodology, termed artificial neural network (ANN), and reported a diagnostic accuracy of 79% in predicting LN metastasis in esophageal cancer.^44^

## Strength and Limitations

The strength of our systematic review lies in its up-to-date unified analysis of esophageal and gastric cancers in different countries. We also identified challenges that will need to be overcome for the technology to be implemented into daily clinical practice. Our study has several weaknesses. First, most of the articles included in the study did not report the specificity or sensitivity of their AI technologies, which prevented a more comprehensive quantitative analysis to achieve a pooled statistic for the diagnostic accuracy of AI. This also prevented the stratification of pooled data based on study intent (diagnostic vs. prognostic). Furthermore, the diagnostic or predictive accuracy of AI depends on several parameters, including the specific AI program or model developed, scanning equipment, image preprocessing, acquisition protocols, and image reconstruction algorithms.

Although there is heterogeneity between the studies, most of the work is limited to a few specific groups that have taken an interest in this field. The majority of the studies are based in Asia, and several of the included papers stem from the work of the same group. Hence, within the same group, the data acquisition and processing techniques are identical but the aims of the study were different and hence merited inclusion. For example, in the studies by Jiang et al., the first study evaluated the use of radiomics and AI in characterizing the tumor microenvironment, while the other study focused on identifying occult metastasis.^30, 31^ Another example are the smaller studies by Giganti et al., each of which separately investigate the response to curative resection and chemotherapy.^27, 28^ Together, these studies shed light on a different aspect of the tumor biology of gastric cancers. In the same vein, we also included some studies with a sample size that was <100. Although small sample sizes lend to a greater degree of variation on the quantitative analysis, these studies were relevant in studying a niche area of treatment response. Larger studies have previously tended to focus on the diagnostic aspects, while other facets such as monitoring for recurrence, response to curative resection and chemotherapy, and tumor heterogeneity are areas that are still in their infancy and hence studied at a smaller level. Furthermore, this emphasizes the paucity of studies of large sample sizes and hints at areas that need further work within the field of AI in esophagogastric cancers.

## Future Directions

Future work should be aimed at the ‘in silico’ bench to bedside translation of these technologies. Although we highlight much promise in these technologies, several factors require evaluation prior to these technologies being employed in routine upper gastrointestinal oncological care:

1. *Use case:* There needs to be early clarification in the lifecycle of these AI devices as to (1) their specific clinical task; (2) potential risk and benefits; (3) whether they are used within either new or existing clinical workflows; and (4) whether they are used independently to diagnose disease/recurrence or as a ‘second reader’ alongside a human clinician. Downstream validation of these systems is dictated by many of these early decisions.
2. *Model development:* The development of these systems are reliant on diverse, large-scale, and well-maintained datasets that are accurately labeled for the purposes of model training and internal validation. Systems created upon small single-center datasets with post hoc labeling rarely perform well when subjected to out-of-set testing.
3. *Validation:* Independent validation of AI systems is crucial, with comparison against expert clinicians to demonstrate either non-inferiority or superiority in diagnostic performance to be undertaken when feasible. Such evaluations require careful study planning, with the need for diverse demographic representation in test datasets in order to assess for bias.
4. *Infrastructural requirements:* Aside from developer considerations, the bottleneck for many contemporary AI products is the end-user adoption and experience. There needs to be careful consideration of the IT infrastructural requirements at hospitals in which these technologies may be reasonably deployed.
5. *Cost effectiveness:* Lastly, although it is assumed that the introduction of AI systems will lead to cost saving across health systems, this requires formal quantification. If deemed not to be financially beneficial, it may be more cost effective to hire diagnostic clinicians, which is the focus on current large-scale studies.

Furthermore, the power of these models is dependent on a large and diverse diet of datasets. At present, the retrospective single-center work available is insufficient and is limited in size, scope and variety. Given that the largest advances in esophagogastric surgery have occurred based on large prospective studies, the advent of ML only calls for further collaborative efforts at an international level to fully reap the potential of this technology.

## Conclusion

AI and radiomics have a huge potential for diagnostic and surveillance of esophageal and gastric cancers. There is currently a paucity of large-scale studies evaluating the usefulness of AI and radiomics in esophageal cancer and the evidence is limited to retrospective studies of small sample sizes. Further progression of its clinical application will require collaborative efforts to generate a large and diverse dataset that can produce an accurate model. This relies on determining the best and most feasible methodology for ML and standardizing this across centers. Hence, further work should focus on these areas.
